# Comparison of Mitochondrial and Antineoplastic Effects of Amiodarone and Desethylamiodarone in MDA-MB-231 Cancer Line

**DOI:** 10.3390/ijms25189781

**Published:** 2024-09-10

**Authors:** Fadi H. J. Ramadan, Balazs Koszegi, Viola B. Vantus, Katalin Fekete, Gyongyi N. Kiss, Balint Rizsanyi, Rita Bognar, Ferenc Gallyas, Zita Bognar

**Affiliations:** 1 Department of Biochemistry and Medical Chemistry, University of Pecs Medical School, 7624 Pecs, Hungary; 2 Szentagothai Research Centre, University of Pecs, 7624 Pecs, Hungary

**Keywords:** apoptosis, mitochondrial dysfunction, mitochondrial drug, cancer cell energy metabolism, drug repositioning

## Abstract

Previously, we have demonstrated that amiodarone (AM), a widely used antiarrhythmic drug, and its major metabolite desethylamiodarone (DEA) both affect several mitochondrial processes in isolated heart and liver mitochondria. Also, we have established DEA’s antitumor properties in various cancer cell lines and in a rodent metastasis model. In the present study, we compared AM’s and DEA’s mitochondrial and antineoplastic effects in a human triple-negative breast cancer (TNBC) cell line. Both compounds reduced viability in monolayer and sphere cultures and the invasive growth of the MDA-MB-231 TNBC line by inducing apoptosis. They lowered mitochondrial trans-membrane potential, increased Ca^2+^ influx, induced mitochondrial permeability transition, and promoted mitochondrial fragmentation. In accordance with their mitochondrial effects, both substances massively decreased overall, and even to a greater extent, mitochondrial ATP production decreased, as determined using a Seahorse live cell respirometer. In all these effects, DEA was more effective than AM, indicating that DEA may have higher potential in the therapy of TNBC than its parent compound.

## 1. Introduction

Amiodarone (AM), developed in 1961, was initially used in Europe, predominantly for treating angina pectoris. In December 1985, the United States Food and Drug Administration (FDA) approved AM for the treatment of life-threatening recurrent hemodynamically unstable ventricular tachycardia and recurrent ventricular fibrillation [1]. The drug prolongs action potential (class III antiarrhythmic effect), and unlike other members of class III, it blocks β-adrenergic receptors, sodium channels, and L-type calcium channels [2,3,4]. However, during long-term, high-dose administration, the drug can have therapy-restricting adverse effects, such as pulmonary toxicity, hepatic injury, worsened arrhythmia, visual impairment, thyroid abnormalities, bradycardia, peripheral neuropathy, photosensitivity, and skin discoloration [5]. The prevalence of the most serious side effect, amiodarone-induced pulmonary toxicity, is estimated to be 5–15% in patients who take ≥400 mg/day and 0.1–0.5% in patients who take up to 200 mg/day [6,7]. Accordingly, the FDA recommends AM therapy only when other antiarrhythmics fail to be effective in the normal therapeutic dose range or are not well tolerated by the patients [1].

AM is metabolized to desethylamiodarone (DEA) by the cytochrome P450 enzyme family of the liver’s detoxifying system and is eliminated predominantly by biliary excretion [8]. A small amount of DEA is also found in the urine [8]. During long-term administration, both AM and DEA rapidly accumulate, especially in adipose tissue and highly vascular organs, such as the lungs, liver, spleen, and lymph nodes [9]. The upper limit of the therapeutic AM plasma concentration was determined to be 5.7 μM [10], since in the majority of cases, yet-unrevealed mechanisms may lead to the development of side effects above these levels. However, tissue accumulation of AM and DEA can result in concentrations 100 to 1000 times higher than the plasma concentration [10]. In addition, there is vast clinical experience with DEA, gathered over more than 50 years of therapeutic use of AM [5]. These findings suggest that DEA can exert sufficiently effective antitumor activity at concentrations not exceeding those measured during the clinical application of AM, making it a readily available agent in cancer therapy.

Adverse effects on mitochondrial function have been identified as a significant mechanism underlying the aforementioned toxic side effects of AM [11,12,13]. Analyses of the chemical moieties within the molecule revealed that the benzofuran structure of AM has been implicated in these effects [12,13]. DEA’s toxicity was reported to correlate with its plasma levels [14] and can be mitigated by the antioxidant N-acetylcysteine [11]. However, the mitochondrial effects of AM have not been associated with increased lipid peroxidation [15], an established marker of oxidative stress [16]. According to our previous findings using isolated mitochondria from rat liver and heart tissues, AM showed protective effects at concentrations up to 10 µM, but harmful effects above that level [17]. In contrast, DEA did not show any protection at low concentrations; rather, it had detrimental effects on mitochondrial function at already lower concentrations than AM [18]. Since anticancer drugs that selectively disrupt the mitochondria of tumor cells have been proposed for the management of treatment-resistant cancers, based on our above-mentioned results, we nominated DEA as a candidate for cancer therapy. This nomination is also supported by our results demonstrating the anti-neoplastic effects of DEA in vitro in various human and rodent cancer cell lines, such as T24 bladder carcinoma [19], HeLa cervix carcinoma [20], B16-F10 melanoma [21], and MCF7 and 4T1 breast cancer lines [22], as well as in vivo in a rodent melanoma lung-metastasis model [21]. In addition, we provided experimental evidence for the involvement of mitochondrial mechanisms in DEA’s anti-neoplastic effects [22,23].

Regarding the anti-cancer potential, we preferred to investigate DEA since its mitochondrial effects were more detrimental than those of AM [17,18], and its tissue accumulation during a chronic treatment exceeded that of the parent compound [8]. However, repositioning an antiarrhythmic drug that is used continuously by a large population for clinical cancer therapy is a much more feasible approach than doing the same for its major endogenous metabolite. Furthermore, the more pronounced mitochondrial effects of DEA compared with those of AM do not necessarily result in superior anti-cancer effects of the former compound. Accordingly, in the present study, we compared the anti-neoplastic and mitochondrial effects of AM and DEA in the MDA-MB-231 human triple-negative breast cancer (TNBC) line.

## 2. Results

### 2.1. Effects of AM and DEA on Mitochondrial Membrane Potential of MDA-MB-231 Cells 

**Figure 1:**
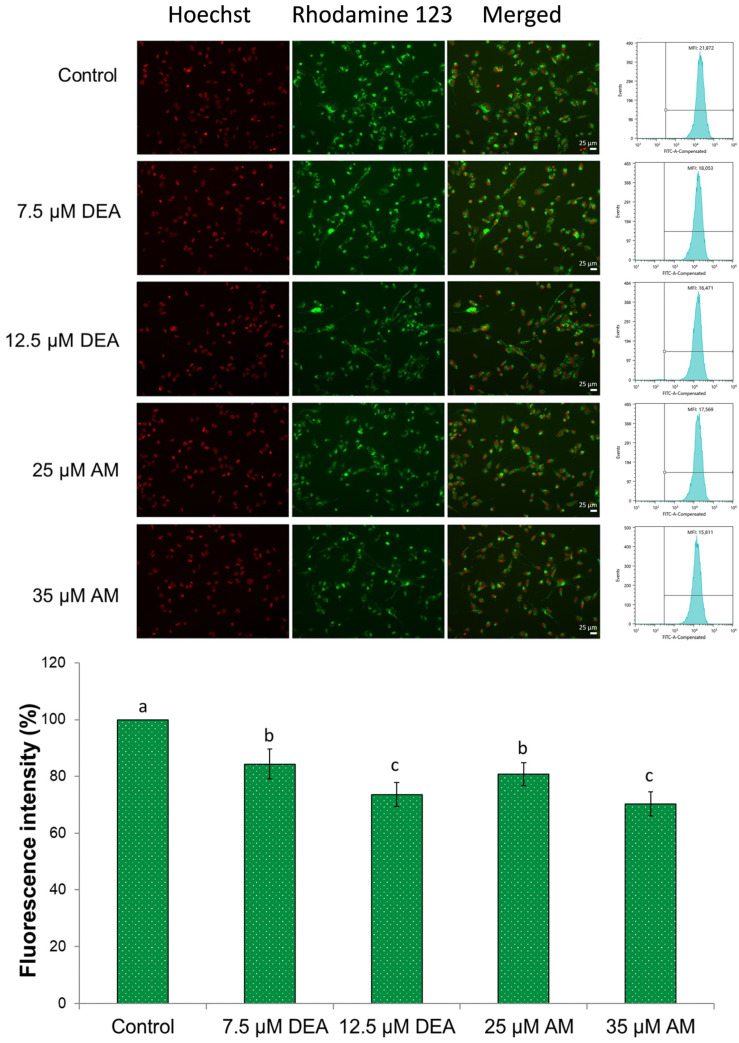
Effects of DEA and AM on ΔΨ_m_ of MDA-MB-231 cells. The cells were treated with 0, 7.5, or 12.5 µM DEA or with 25 or 35 µM AM for 6 h before loading them with R-123 dye and taking fluorescent microscopy images, as well as flow cytometry readings. The nuclei were counterstained with Hoechst 33342 (Hoechst). The results are presented as representative microscopy images of the same field in the red and green channels, as well as flow cytometry histograms of cells. The scale bar represents 25 µm. The horizontal and vertical axes of the histograms represent the fluorescence intensity of R-123 on a logarithmic scale of 10^1^–10^6^ arbitrary units and event number on a linear scale of 0–400, respectively. The bar diagram shows the mean fluorescent intensity normalized to the fluorescent intensity of control, mean ± standard error of the mean (SEM) of three independent experiments. The letters above the bars denote groups significantly (*p* < 0.05) different from the others.

Mitochondrial membrane potential (ΔΨm) provides the driving force for several mitochondrial functions, including ATP synthesis, ion and protein transport, and mitochondrial quality control [24]. Also, ΔΨm is being used as an early apoptotic marker [25]. We intended to determine whether AM affects ΔΨm in living cells in a similar way as we detected that with DEA in our previous experiments [22]. For this analysis, we used a fluorescent dye with low toxicity, rhodamine 123 (R-123), which, due to its cationic structure, can enter and accumulate in healthy mitochondria, depending on the membrane potential [26]. The reduction or loss of membrane potential results in low dye accumulation and the membrane being unable to retain R-123. We treated MDA-MB-231 cells with DEA or AM for 6 h before loading them with R-123 and taking fluorescence microscopy images, as well as flow cytometry readings. Perfectly in line with our previous results on isolated mitochondria, in live MDA-MB-231 cells, DEA decreased ΔΨ more effectively than AM in a concentration-dependent manner (Figure 1).

### 2.2. Effects of AM and DEA on Mitochondrial Network Dynamics in MDA-MB-231 Cells

**Figure 2:**
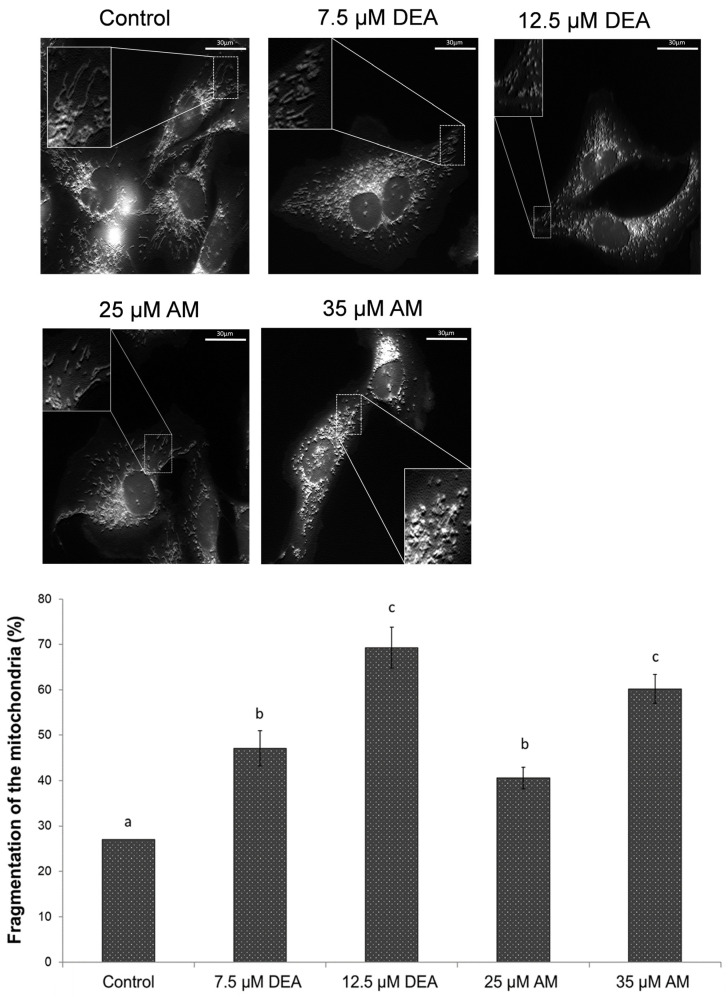
Effects of DEA and AM on mitochondrial network dynamics in MDA-MB-231 cells. The cells were treated with 0, 7.5, or 12.5 µM DEA or with 25 or 35 µM AM for 6 h before loading them with MitoTracker red dye and taking fluorescence microscopy images. Mitochondrial fragmentation was determined as described earlier [29]. The data are presented as representative images and as the percentage of fragmented mitochondria, mean ± SEM of three independent experiments. The scale bar represents 30 µm. The letters above the bars denote groups significantly (*p* < 0.05) different from the others.

The dynamic properties of mitochondria play a critical role in optimizing mitochondrial function, especially energy production [27], while their alterations have been implicated in various disorders, including cancer [28]. Since ΔΨm is a key regulator of the mitochondrial fusion–fission balance [27,28], we investigated whether AM affects mitochondrial fragmentation in a similar way as previously observed with DEA [19]. MDA-MB-231 cells were treated with DEA or AM for 6 h, similar to the ΔΨm analysis; then, cells were loaded with MitoTracker Red to visualize the mitochondria and take fluorescence microscopy images. Both DEA and AM induced mitochondrial fragmentation in MDA-MB-231 cells in a concentration-dependent manner (Figure 2). However, AM had to be applied in a higher concentration than DEA to achieve the same extent of mitochondrial fragmentation (Figure 2). 

**Figure 3:**
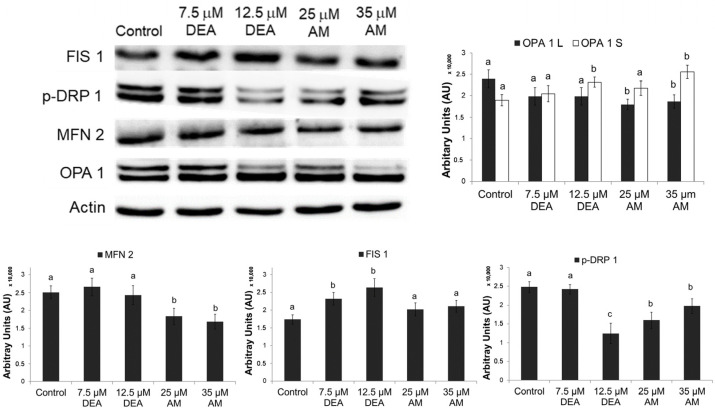
Effects of DEA and AM on proteins regulating the mitochondrial fusion-fission process. MDA-MB-231 cells were treated with 0, 7.5, or 12.5 µM DEA or with 25 or 35 µM AM for 6 h, then homogenates of the cells were subjected to immunoblot analysis. The data are presented as representative blots and as pixel densities of protein bands, mean ± SEM of three independent experiments. The lower-case letters above the bars denote groups significantly (*p* < 0.05) different from the others of the same set. OPA1 L and S denote the long (active) and short (cleaved, inactive) forms of the protein.

Mitochondrial fusion and fission processes are mediated by large GTPases, which are regulated by various mechanisms, including phosphorylation and proteolytic cleavages [27,28]. To investigate the proteins involved in these processes, we performed immunoblot analysis on homogenates of MDA-MB-231 cells treated identically to the fragmentation experiments. We found that DEA treatment elevated the steady-state levels of mitochondrial fission 1 protein (FIS1) (Figure 3), the indirect regulator of mitochondrial fission, by recruiting dynamin-related protein 1 (DRP1), the key large GTPase responsible for the process. AM increased the levels of FIS1; however, this increase was not statistically significant (Figure 3). Also, AM and, to a greater extent, DEA reduced the protein kinase A-mediated inhibitory phosphorylation of DRP1-Ser^637^ (Figure 3). Furthermore, AM decreased mitofusin 2 (MFN2) levels and increased the cleavage of optic atrophy protein 1 (OPA1), the large GTPase responsible for the fusion of the inner and outer mitochondrial membranes (Figure 3). Interestingly, DEA treatment did not affect MFN2 levels and the OPA1 cleavage-inducing effect of DEA was somewhat inferior to that of AM (Figure 3). These results are consistent with the elevated mitochondrial fragmentation (Figure 2) caused by the two drugs and indicate that DEA likely acts more on the fission while AM acts more on the fusion side of the fission–fusion equilibrium (Figure 3).

### 2.3. Effects of AM and DEA on Mitochondrial Permeability Transition in MDA-MB-231 Cells 

**Figure 4:**
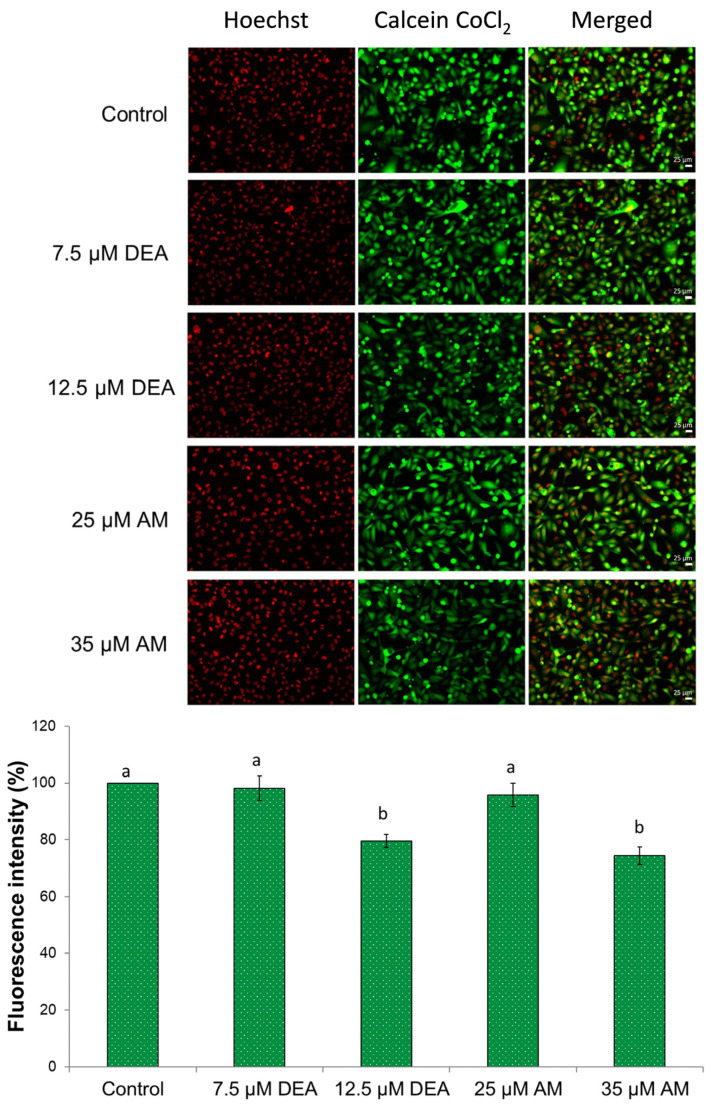
Effects of DEA and AM on mPT in MDA-MB-231 cells. The cells were treated with 0, 7.5, or 12.5 µM DEA or with 25 or 35 µM AM for 6 h before loading them with calcein + CoCl_2_ and taking fluorescent microscopy images. The nuclei were counterstained with Hoechst. The data are presented as representative merged images of the same field in the red and green channels, and as corrected total cell fluorescence (CTCF) normalized to the fluorescence intensity of the control, mean ± SEM of three independent experiments. The scale bar represents 25 µm. The lower-case letters above the bars denote groups significantly (*p* < 0.05) different from the others.

The activation of the mitochondrial permeability transition (mPT) pore, a megachannel that allows the unregulated passage of molecules up to 1.5 kDa between the cytoplasm and the mitochondrial matrix [30], has been suggested as a promising strategy for improving anticancer therapies [31]. Since both AM and DEA were demonstrated to trigger mPT in isolated mitochondria [18], we were interested in whether they had any such effect in MDA-MB-231 cells. Accordingly, we treated MDA-MB-231 cells with 0–35 µM AM or 0–12.5 µM DEA for 6 h and assessed mPT in the cells using the calcein O,O′-diacetate tetrakis(acetoxymethyl) ester (calcein)-Co^2+^ quenching fluorescent microscopy technique, as previously described [32]. In complete agreement with our previous results in isolated mitochondria [18], 35 µM AM or 12.5 µM DEA induced significant mPT, nearly to the same extent, in MDA-MB-231 cells (Figure 4). Therefore, similar to the ΔΨ_m_ analysis, DEA proved to be more effective than AM.

### 2.4. Effects of AM and DEA on Intracellular Calcium Levels ([Ca^2+^]_i_) of MDA-MB-231 Cells

**Figure 5:**
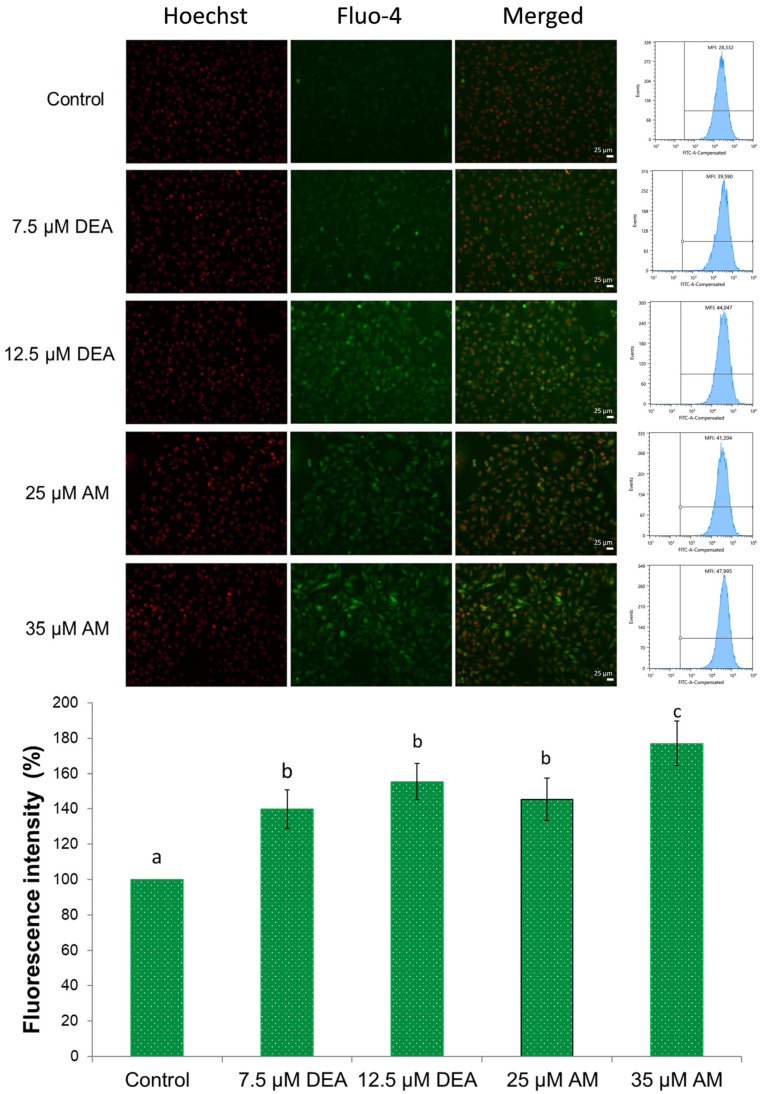
Effects of DEA and AM on [Ca^2+^]_i_ in MDA-MB-231 cells. The cells were treated with 0, 7.5, or 12.5 µM DEA or with 25 or 35 µM AM for 3 h before loading them with Fluo-4AM dye and taking fluorescent microscopy images, as well as flow cytometry readings. The nuclei were counterstained with Hoechst. The results are presented as representative microscopy images of the same field in the red and green channels, as well as flow cytometry histograms of cells. The scale bar represents 25 µm. The horizontal and vertical axes of the histograms represent the fluorescence intensity of Fluo-4AM on a logarithmic scale of 10^1^–10^6^ arbitrary units and event number on a linear scale of 0–300, respectively. The bar diagram shows the fluorescence intensity normalized to the fluorescence intensity of the control, mean ± SEM of three independent experiments. The letters above the bars denote groups significantly (*p* < 0.05) different from the others.

The deregulation of Ca^2+^ homeostasis has long been associated with various cancer hallmarks, such as malignant transformation, tumor progression, and resistance to therapy [33,34]. Furthermore, increased mitochondrial Ca^2+^ uptake is a potent inducer of mPT [30,35]. Accordingly, we were interested in whether AM or DEA treatment affects [Ca^2+^]_i_. We used the fluorescent Ca^2+^ probe Fluo-4AM to assess [Ca^2+^]_i_ in MDA-MB-231 cells after treating them with 0–35 µM AM or 0–12.5 µM DEA in the presence of 3 mM CaCl_2_ for 3 h. Both compounds increased [Ca^2+^]_i_ in a dose-dependent manner, and even their lower concentrations (7.5 µM and 25 µM, respectively) were able to significantly elevate [Ca^2+^]_i_ to approximately the same extent (Figure 5).

### 2.5. Effect of AM and DEA on Energy Metabolism of MDA-MB-231 Cells

**Figure 6:**
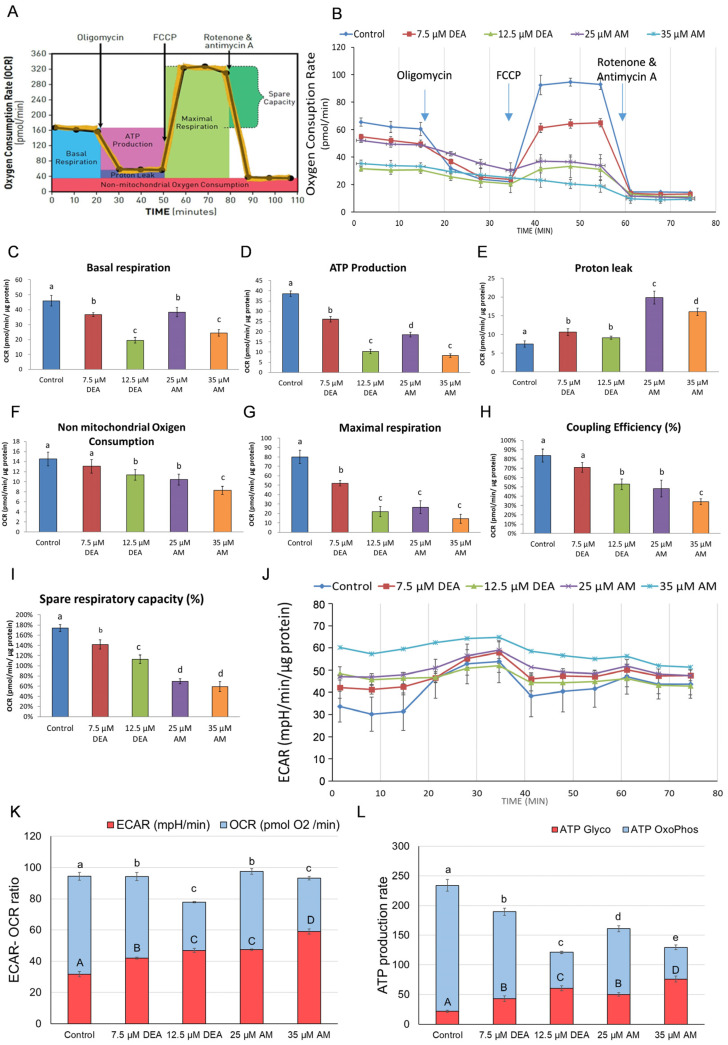
Effects of DEA and AM on energy metabolism of MDA-MB-231 cells. The cells were treated with 0 to 12.5 µM DEA or 0 to 35 µM AM for 6 h before recording OCR and ECAR for 75 min. The F_o_F_1_ ATPase inhibitor oligomycin, the uncoupler FCCP, and the respiratory inhibitors rotenone and antimycin A were added at 15, 35, and 55 min of the recording. The experimental protocol and the scheme for calculating the various respiratory parameters are shown in panel (**A**). Data are presented as representative original recordings (**B**,**J**) and as automatically calculated parameters (**C**–**I**); mean ± SD of three independent experiments running in two replicates. OCR and ECAR data were normalized to mg protein content. OCR-ECAR ratios (**K**) and ATP production rates (**L**) were calculated from the raw data of panels (**B**,**J**) according to [40]. The upper- and lower-case letters above the bars denote groups significantly (*p* < 0.05) different from the others. In panels (**K**,**L**), blue and red bars denote oxidative phosphorylation and glycolysis, respectively.

Mitochondrial metabolism has been suggested as a promising target for the therapy of cancers with limited treatment options, such as TNBC [36,37]. To demonstrate the inherent potential of DEA in this regard, we showed that this agent reduced various parameters of oxidative phosphorylation in BC lines [22]. In the present study, we investigated whether its parent compound, AM, may also possess such an effect. To this end, we monitored the cellular oxygen consumption rate (OCR) and extracellular acidification rate (ECAR) after treating MDA-MB-231 TNBC cells with 0–35 µM AM or 0–12.5 µM DEA for 6 h. To evaluate ATP production, oligomycin, an F_o_F_1_ ATPase inhibitor, was added to the system after 15 min of recording basal respiration. The maximal respiratory rate was determined by adding carbonyl cyanide 4-(trifluoromethoxy) phenylhydrazone (FCCP), a mitochondrial uncoupling agent. Mitochondrial respiration was then completely blocked by the administration of the Complex I and Complex III inhibitors rotenone and antimycin A, allowing the calculation of proton leak and non-mitochondrial oxygen consumption (Figure 6A). In agreement with previous results [22], DEA decreased the various parameters of oxidative phosphorylation in MDA-MB-231 cells and AM induced very similar, although less pronounced, changes (Figure 6B–I). Regarding the most important functional parameters, 12.5 µM DEA reduced ATP production (Figure 6D) and coupling efficiency (Figure 6H) to about the same extent as 25 µM AM. 

The time courses of ECAR for the different treatments (Figure 6J) were inversely proportional to the corresponding OCR time courses (Figure 6B). Accordingly, both AM and DEA increased ECAR at the expense of OCR in a concentration-dependent manner (Figure 6K). OCR and ECAR readings were converted to rates of ATP production by oxidative phosphorylation and glycolysis, respectively, using the principle of Mookerjee et al. [38,39], and adapting the method of Desousa et al. [40] to our experimental conditions. The results indicate that DEA and AM decreased the total ATP production (Figure 6D) of MDA-MB-231 cells by about 50% while shifting the balance of oxidative and fermentative energy production from about 10:1 to 1:1 (Figure 6L).

### 2.6. Anti-Neoplastic Effects of AM and DEA in MDA-MB-231 Cells

**Figure 7:**
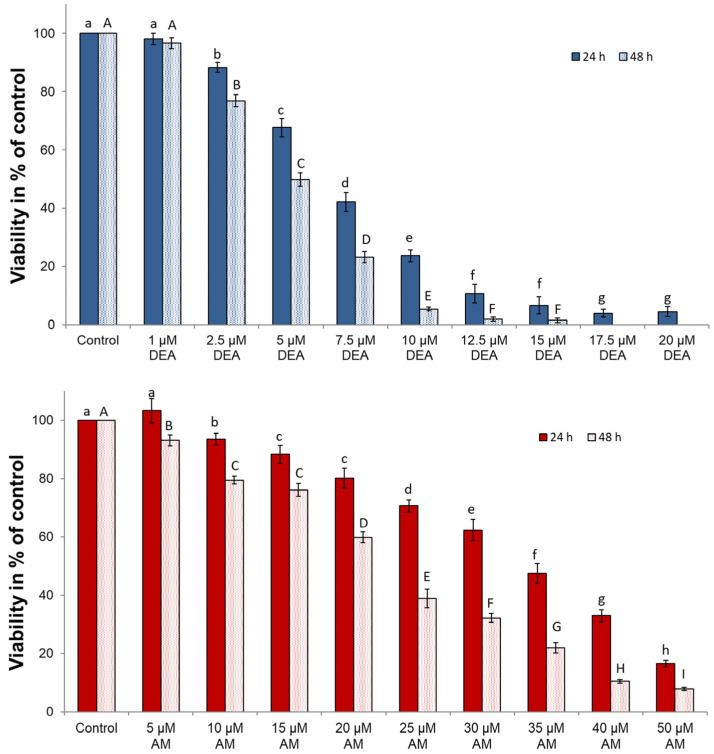
Effects of DEA and AM on the viability of MDA-MB-231 cells. We treated the MDA-MB-231 cells with 0–20 µM DEA or 0–50 µM for 24 h (dark bars) or 48 h (light bars) before measuring their viability using the SRB assay. We presented viability as the percentage of the untreated control, means ± SEM of five independent experiments. The upper and lower-case letters above the bars denote groups significantly (*p* < 0.05) different from the others.

We compared how the two drugs influenced the viability and apoptosis of MDA-MB-231 cells to investigate whether the difference in their mitochondrial effects is reflected in their anti-neoplastic potential. Cells were treated with 0 to 50 µM AM or 0 to 20 µM DEA for 24 or 48 h before their viability was determined by using the sulforhodamine B (SRB) assay recommended to determine the cytotoxicity of mitochondria-targeting substances [41]. Both compounds effectively decreased the viability of MDA-MB-231 cells in a concentration-dependent manner (Figure 7). However, DEA was more effective than its parent compound, and it completely eradicated the MDA-MB-231 cells at a concentration of 17.5 µM over a 48 h incubation period (Figure 7).

**Figure 8:**
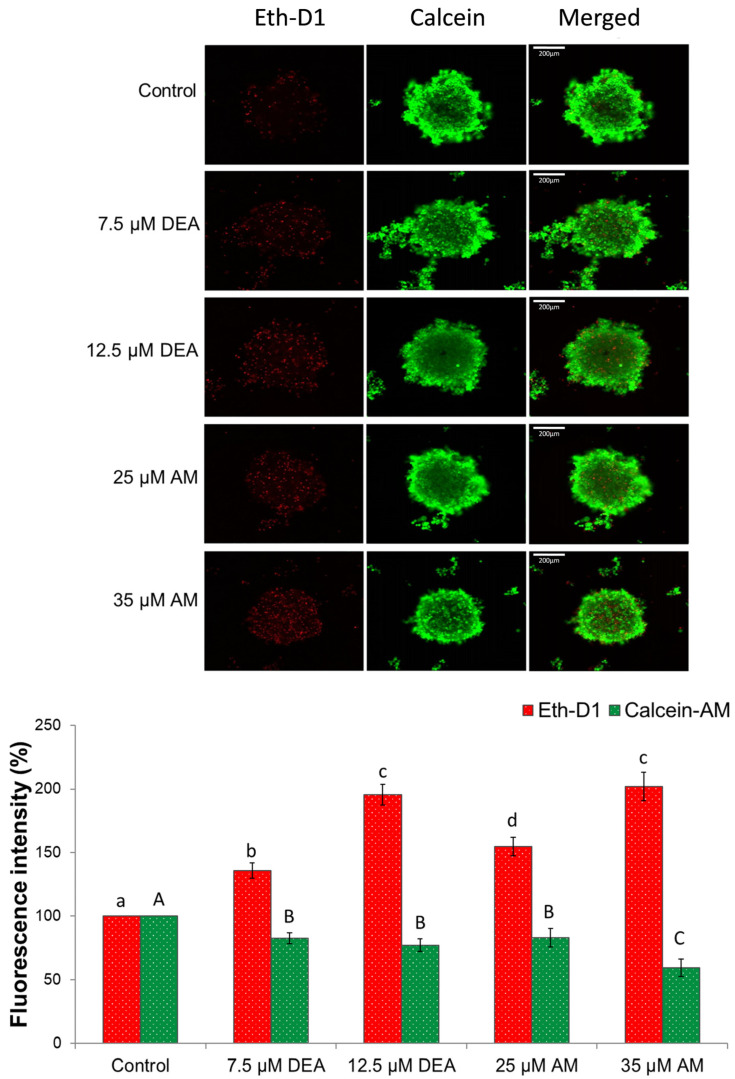
Effects of DEA and AM on the viability of MDA-MB-231 spheroids. We treated the spheroid cultures with 0, 7.5, or 12.5 µM DEA or with 25 or 35 µM AM for 72 h before adding calcein and ethidium homodimer (ETHD) to the medium. Images were captured in the green and red channels of a fluorescence microscope. The results are presented as representative images and bar diagrams of three independent experiments. For the latter, CTCFs were normalized to the total protein content of permeabilized spheroids and were expressed as the percentage of the untreated cultures mean ± SEM. The scale bar represents 200 µm. The upper- and lower-case letters above the bars denote groups significantly (*p* < 0.05) different from the others of the same set.

Compared with conventional culture conditions, three-dimensional culture systems of cancer cells exhibit cell–cell interactions, gene expression, and signaling pathway profiles that better resemble physiological conditions and provide a more reliable platform for studying anti-neoplastic compounds [42]. Accordingly, we studied the effects of AM and DEA on the viability of MDA-MB-231 spheroid cultures. Similarly to the results of the simple viability assay, both compounds increased the ratio of dead cells in a concentration-dependent manner (Figure 8). However, in the experiments, DEA proved to be more effective than its parent compound, exerting approximately the same effect at much lower concentrations (Figure 8).

### 2.7. Apoptosis-Inducing Effects of AM and DEA in MDA-MB-231 Cells 

**Figure 9:**
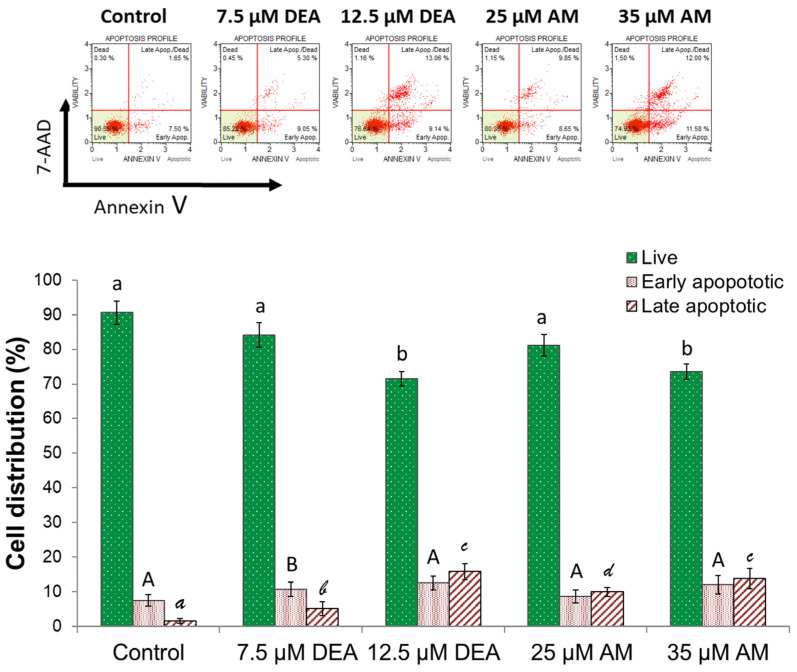
Effect of DEA and AM on apoptosis in MDA-MB-231 cells. We treated the MDA-MB-231 cells with 0, 7.5, or 12.5 µM DEA or with 25 or 35 µM AM for 6 h before determining the type of cell death using flow cytometry followed by double-staining with FITC-Annexin V and 7-AAD. The results are presented as representative scatter plots and bar diagrams of three independent experiments. The x (Annexin V) and y (viability) axes of the scatter plots indicate FITC-Annexin V and 7-AAD fluorescence intensities, respectively. In the bar diagrams, live (green bars), early (middle bars), and late (striped bars) apoptotic cells are expressed as the percentage of the total cell number mean ± SEM. The different characters above the bars denote groups significantly (*p* < 0.05) different from the others of the same set.

The collapse of ΔΨm, as well as mPT, may lead to apoptotic cell death [30]. The mitochondrial effects of the investigated drugs were significant, even after a 6-h incubation period. We studied whether these effects could have led to significant apoptotic cell death during this relatively short period of time. Accordingly, MDA-MB-231 cells were treated exactly as in the ΔΨm and mPT experiments, then double-stained with fluorescein isothiocyanate (FITC)-labeled Annexin V and 7-aminoactinomycin D (7-AAD), and analyzed by flow cytometry. The 6 h exposure reduced cell viability by 5–15%, accompanied by a corresponding increase in the number of apoptotic cells (Figure 9). DEA proved to be more effective again, causing a similar level of apoptosis at 12.5 µM as AM at 35 µM. (Figure 9).

**Figure 10:**
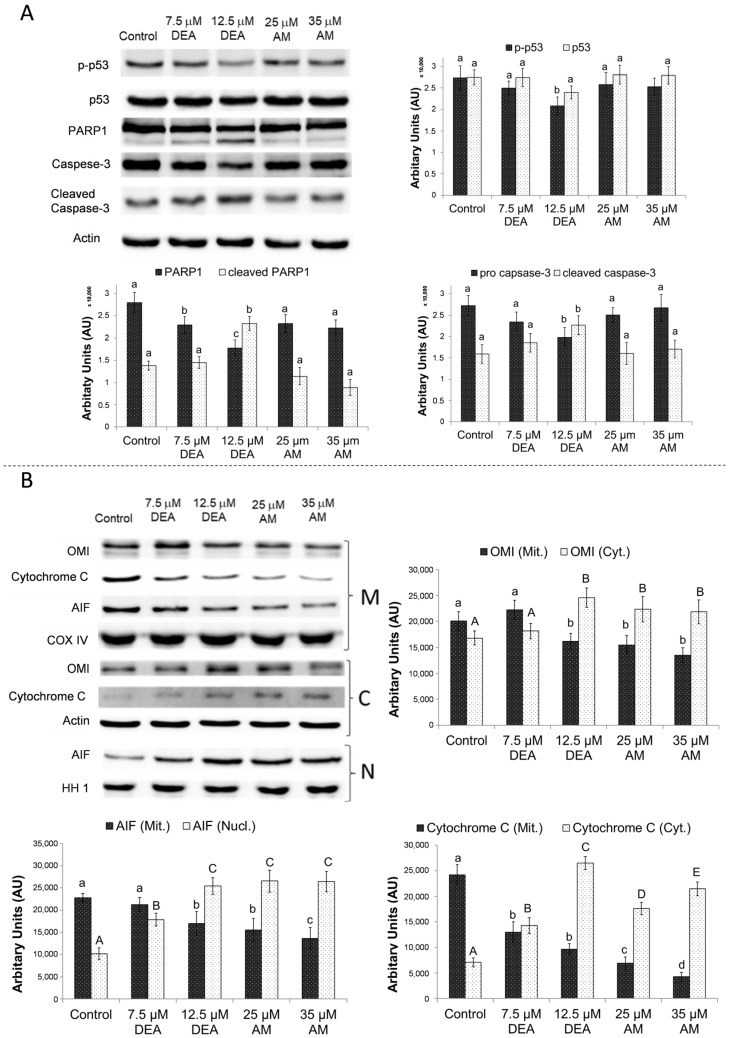
Effects of DEA and AM on apoptosis markers in MDA-MB-231 cells. We treated the MDA-MB-231 cells with 0, 7.5, or 12.5 µM DEA or with 25 or 35 µM AM for 24 h before homogenization (**A**) or isolation of nuclear (N), mitochondrial (M) and cytosolic (C) extracts by subcellular fractionation (**B**). We assessed the levels of p53 phosphorylation, caspase 3- and PARP1-cleavage (**A**), and the release of OMI, AIF, and cytochrome C from the mitochondria (**B**) by immunoblotting. Actin (whole cell and cytosol), cytochrome oxidase (COX IV; mitochondrium), and histone H1 (HH1; nucleus) were used as loading controls. The results are presented as representative blots and pixel densities of the bands, mean ± SEM of three independent experiments. The upper- and lower-case letters above the bars denote groups significantly (*p* < 0.05) different from the others of the same set.

After treating the cells according to the same protocol for 24 h, we also studied the effects of these compounds on various markers of apoptosis. We performed immunoblotting to determine transformation-related protein 53 (p53) phosphorylation, caspase-3 cleavage, and poly(ADP-ribose) polymerase 1 (PARP1) cleavage [43] from whole-cell homogenates. Mitochondrial, nuclear, and cytosolic fractions of identically treated cells were also isolated and the mitochondrial release of cytochrome C, high-temperature requirement protein A2 (OMI), and apoptosis-inducing factor (AIF) into the cytosol and nucleus was measured by immunoblotting [44]. The substances did not seem to significantly affect the steady-state levels or the phosphorylation of p53 (Figure 10A). DEA, however, induced caspase-3 and PARP1 cleavage in a concentration-dependent manner, while AM did not affect these processes (Figure 10A). On the other hand, both substances caused OMI, AIF, and cytochrome C release from the mitochondria in a concentration-dependent manner, and interestingly, the effect of AM on these markers was stronger than that of DEA (Figure 10B). 

### 2.8. Effects of AM and DEA on Wound-Healing in MDA-MB-231 Cells 

**Figure 11:**
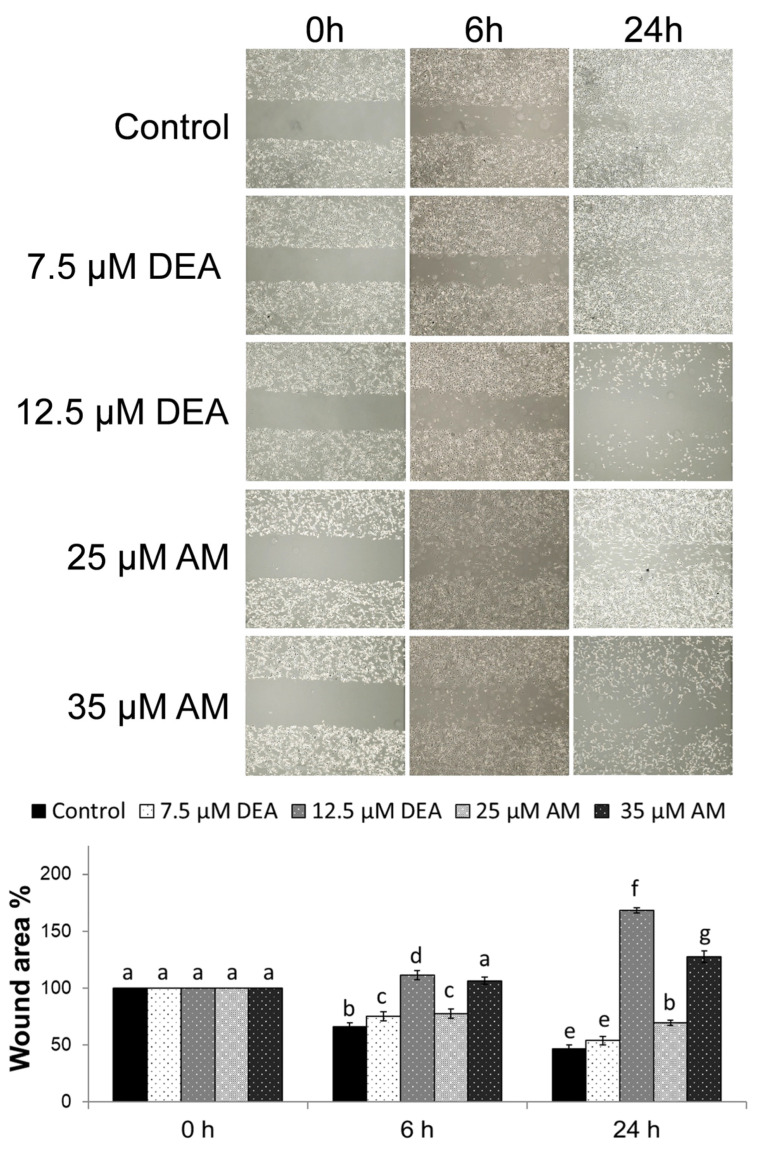
Effects of DEA on wound healing in MDA-MB-231 cell line. A wound was inflicted onto semi-confluent cultures of MDA-MB-231 cells and the cultures were exposed to 0, 7.5, or 12.5 µM DEA or 25 or 35 µM AM for 12 h. The data are presented as representative images of the wounds taken at 0, 6, and 24 h, and the wound area is expressed as the percentage of untreated plates at the 0 h time-point, mean ± SEM of two independent experiments running in duplicates. The letters above the bars denote groups significantly (*p* < 0.05) different from the others of the same set.

Cell migration, a critical component of cancer invasiveness, is frequently evaluated by the wound-healing assay [45]. We introduced a wound into semi-confluent monolayer cultures of MDA-MB-231 cells and treated the cultures with 0, 7.5, or 12.5 µM DEA, or with 25 or 35 µM AM for 6 or 24 h. Consistent with its TNBC phenotype, MDA-MB-231 cells migrated intensely and, within 24 h, almost completely closed the wound. This was significantly delayed by both compounds in a concentration-dependent manner (Figure 11). At higher concentrations, the compounds caused wound exacerbation by killing the cells (Figure 11). DEA again proved to be more effective than AM and showed stronger effects at lower concentrations (Figure 11).

## 3. Discussion

In this study, we compared the mitochondrial and antineoplastic effects of the anti-arrhythmic drug AM and its major metabolite DEA to evaluate their potential in cancer therapy. Previously, DEA was demonstrated to exert more detrimental mitochondrial effects than AM, both in isolated mitochondria and cultured cell lines [17,18]. In addition, DEA exhibited potent anti-proliferative properties in various cancer lines, as well as in an in vivo metastasis model at concentrations within the anti-arrhythmic therapeutic range [19,20,21,22,23]. Furthermore, during in vivo AM treatment, DEA is always produced by the detoxification system of the liver without being further metabolized [46]. The simultaneous presence of AM and DEA has been shown to cause additive cytotoxicity that may significantly contribute to the serious side effects caused by AM [47]. Based on its stronger cytotoxic effect compared with AM, we have proposed DEA as a potential anti-cancer drug candidate. However, the aim of this study was to contribute to the repositioning of AM, a drug already approved for human therapy, a process less complicated than the introduction of its metabolite DEA as a new drug for cancer therapy. 

Cancers of aggressive phenotypes such as TNBC face a seemingly impossible challenge of rapid proliferation in an environment where nutrient and oxygen availability is low [48]. They overcome this challenge by re-directing a substantial amount of cellular glucose for the production of various precursors of nucleotide and triglyceride biosynthesis [49]. The energy demand of such cancer cells is maintained by lactic acid fermentation, even in the presence of oxygen, which is provided by the hypoxia-inducible factor-1 (HIF-1)-mediated uncoupling of the tricarboxylic acid cycle from glycolysis [50]. However, drug-resistant metastasizing cancer cells can especially revert from reductive to oxidative metabolism [51]. Thus, most high-clinical-grade cancer types produce energy mainly by mitochondrial oxidative phosphorylation [52,53]. Accordingly, the finely balanced metabolism of these robust tumor cells is regarded as a therapeutic target, and drugs capable of disrupting the balance between their oxidative and reductive metabolism are considered to have great potential in the clinical management of these aggressive tumor types [37,54,55]. 

Various mitochondrial processes, like the tricarboxylic acid cycle and the electron transport chain, the integrity of the mitochondrial membranes, and the regulation of mitochondrial network dynamics, were suggested as targets for cancer therapy [37,54,56,57,58]. Although many compounds are in the experimental, Phase I, or Phase II stages of their development, only a few have been approved for clinical practice; inhibitors of isocitrate dehydrogenase, α-ketoglutarate dehydrogenase, pyruvate dehydrogenase, Complex I and Complex IV, and a Bcl-2 antagonist [56,58]. They have very different toxicities, mechanical properties, and approved applications ranging from leukemias to solid tumors [56,58]. Accordingly, it is challenging to compare their efficacy on a molecular basis. However, they all show significant antineoplastic effects in their respective therapeutic windows [56,58]. In the same way, at physiologically relevant concentrations, we found both AM and DEA to be effective against MDA-MB-231 TNBC cells according to viability studies on monolayers (Figure 7) and spheroids (Figure 8), and wound-healing experiments (Figure 11). They included apoptotic cell death, as demonstrated both by the fluorescent staining of the phosphatidylserine residues in the outer face of the cell membrane (Figure 9) and by assessing various apoptotic markers, such as the cleavage of PARP1 and caspase-3, and release of AIF and cytochrome C from the mitochondria (Figure 10). In all these experiments, equimolar DEA was more effective than AM.

Mitochondria play a pivotal role in regulating cancer cell survival, which depends on ATP production, ROS generation, and the intrinsic apoptotic pathway [59]. These pathways are interdependent. ΔΨm, together with the chemical potential of the hydronium ion gradient across the mitochondrial inner membrane, represents the driving force for mitochondrial ATP synthesis. However, the operation of the electron transport chain during oxidative phosphorylation produces a significant amount of ROS, which can damage the mitochondrial membrane systems, subsequently decreasing ATP and further increasing ROS production [37]. Compromising the integrity of the outer mitochondrial membrane initiates apoptosis via the release of pro-apoptotic inter-membrane proteins, such as cytochrome C, AIF, and endonuclease G, while permeabilizing the inner membrane results in ΔΨm loss and mPT [60]. Additionally, increased [Ca^2+^]_i_, which could result from ROS accumulation, can also trigger mPT, eventually leading to necrotic cell death [35]. Similarly to their previous action on isolated liver and rat mitochondria [17,18], AM, and more efficiently DEA, reduced ΔΨm (Figure 1), elevated [Ca^2+^]_i_ (Figure 5), and induced mPT (Figure 4) in a concentration-dependent manner, indicating that their anti-neoplastic property was based on their mitochondrial effects. 

Regarding mitochondrial network dynamics, the continuous fusion and fission of mitochondria, is essential for mitochondrial biogenesis, quality control, retrograde signaling, and adjusting cellular energy production to metabolic demands [27,28]. The large GTPases, MFN 1 and 2 and OPA1 mediate fusion, while DRP1 is responsible for fission [27]. DRP1 is recruited to the mitochondria by Fis1 and is regulated by phosphorylation [27]. In pathological situations, fusion is often prevented by ΔΨm being too low, resulting in the accumulation of fragmented mitochondria, which are more damage-prone and eliminated by mitophagy [28]. Accordingly, mitochondrial fragmentation and reduced mitochondrial copy numbers are frequently found in many cancer types, such as breast, colon, and hepatocellular carcinomas, astrocytomas, and prostate cancer [28]. As demonstrated in Figure 2 and Figure 3, both AM and DEA induced mitochondrial fragmentation in a concentration-dependent manner in the MDA-MB-231 TNBC line. Again, equimolar DEA was more effective than AM (Figure 2).

In addition to energy production, augmented aerobic glycolysis promotes tumorigenesis in several other ways. Most importantly, intermediates of glycolysis can also be utilized in anabolic pathways to synthetize the amino acids, nucleotides, and lipids necessary for cancer cell proliferation [60]. Furthermore, glycolysis promotes epithelial-to-mesenchymal transition (EMT) by activating EMT factors and providing an acidic environment via lactate production [61]. Also, lactate secreted into the tumor environment helps to evade immune surveillance by inhibiting cytotoxic T cells and inducing M2 polarization of macrophages [59]. On the other hand, mitochondrial energy production is maintained or even increased in many cancer cells, and they manage to oxidize carbon sources, such as glutamine, fatty acids, and lactate, besides glucose [60,61]. This metabolic reprogramming, which provides a survival advantage for cancer cells in an environment poor in nutrients and oxygen, such as the core of solid tumors, can also be targeted for therapeutic purposes [37,52,54,55,57,60,61]. Accordingly, drugs affecting glycolysis and/or oxidative phosphorylation could have therapeutic value [37,52,54,55,57,60,61]. We demonstrated that AM and DEA can be considered such therapeutic tools, since AM and more effectively DEA suppressed both glycolytic and mitochondrial ATP production in a concentration-dependent manner (Figure 6).

As it has already been mentioned, the mitochondrial processes we analyzed in this study are interrelated. Accordingly, it is difficult to identify the molecular target of AM and/or DEA. For example, Venetoclax, a B-cell lymphoma (Bcl) homology domain-3 (BH3) mimetic, prevents the complex formation between Bcl2 and proapoptotic BH3-only proteins, thereby destabilizing the outer mitochondrial membrane and inducing apoptosis in cancer cells by mitochondrial swelling, depolarization and fragmentation [62]; findings very similar to those we obtained with AM and DEA. However, according to our previous studies, AM and DEA also exerted effects on isolated mitochondria [17,18]; hence, the molecular target of these drugs should be on the surface or inside of the mitochondria, the identification of which requires further investigations.

Previously, in isolated mitochondria, low concentrations of AM inhibited, rather than induced, mPT, while DEA induced it in a concentration-dependent manner in the whole concentration range [17]. Additionally, a low concentration of AM, but not DEA, was found to protect post-ischemic isolated Langendorff-perfused hearts, the effect of which was attributed partially to the inhibition of mPT by the former compound [18]. Perfectly in line with these findings, in the present study, equimolar DEA demonstrated more pronounced mitochondrial and anti-neoplastic effects than AM. Although DEA is implicated in the toxic side-effects of a prolonged high-dose AM therapy [5,11,12,13,14,15], at a therapeutic dosage, these side-effects are mostly tolerated by patients [5]. Therefore, the more pronounced toxic effects of DEA than AM could be beneficial in cancer treatment, since lower concentrations of the former can be utilized.

## 4. Materials and Methods

### 4.1. Materials

The triple-negative breast cancer MDA-MB-231 cell line was purchased from the American Type Culture Collection (Manassas, VA, USA). The DEA was a gift from Professor Andras Varro (Department of Pharmacology and Pharmacotherapy, University of Szeged, Szeged, Hungary). The protease and phosphatase inhibitor cocktail and other chemicals for cell culture were purchased from Sigma-Aldrich Kft (Budapest, Hungary). The following primary antibodies were used: anti-Fis1, anti-phospho-DRP1, anti-MFN2, anti-OPA1, anti-p53, anti-phospho-p53, anti-PARP1, anti-caspase-3, anti-OMI, anti-AIF, anti-COX IV, anti-histone H1, and anti-actin (1:2000).

### 4.2. Cell Cultures

MDA-MB-231 cells were cultured and maintained as monolayer-adherent cultures in Leibovitz’s L-15 medium supplemented with 10% fetal bovine serum (FBS) under standard conditions. Depending on the experiment’s type, the cells were plated in different plates at different densities.

### 4.3. Cell Viability Assay

To assess the cytotoxicity of the drugs on MDA-MB-231 cells, the SRB assay (Sigma-Aldrich, St. Louis, MO, USA) was performed. MDA-MB-231 cells were seeded onto 96-well plates at a starting density of 8 × 10^3^ cells/well in quintuplicate (five replicate wells per sample) overnight. On the second day, cells were treated with 0–20 µM DEA and 0–50 µM AM for 0, 24, and 48 h. Following each treatment, the plates were washed with 1× phosphate-buffered saline (PBS) and fixed in 100 µL of chilled 10% trichloroacetic acid solution (TCA) at 4 °C for 30 min. Then, the plates were washed five times with deionized water and allowed to dry overnight at room temperature. An amount of 70 µL of 0.4% SRB (Sigma-Aldrich, St. Louis, MO, USA) dye prepared in 1% acetic acid was added to the wells and incubated at room temperature for 30 min. After that, the dye was removed and the plates were washed 5 times with 1% acetic acid, and then allowed to dry for a few hours at room temperature. When fully dried, 200 µL of 10 mM tris(hydroxymethyl)aminomethane base was added to each well and incubated on a plate shaker for 30 min. Absorbance was measured at 560 and 600 nm in parallel with a GloMax^®^-Multi Instrument (Promega, Fitchburg, WI, USA), OD_600_ was subtracted as a background from the OD_560_. The experiment was repeated 5 times.

### 4.4. Apoptosis Assay

To detect the average difference between live and apoptotic cells within DEA or AM treated MDA cells, a *MUSE Annexin V & Dead cell* Kit (Luminex Corporation, Austin, TX, USA) was used. The experiments were conducted according to the manufacturer’s protocol. MDA cells were cultured in 6-well plates (1.5 × 10^5^ cells/well) in duplicate overnight. On the second day, the cells were treated with 0, 7.5, or 12.5 µM DEA, or 25 or 35 µM AM for 6 h. After treatment, the cells were harvested and diluted in their medium. An amount of 100 µL of Annexin V reagent was added to the samples, followed by 20 min of incubation in a dark room at room temperature. A total of 5000 single-cell events were measured per sample using a MUSE Cell Analyzer device (Millipore, Burlington, MA, USA). The experiment was repeated twice.

### 4.5. Migration Assay

To study the effects of different drug concentrations on two-dimensional MDA cells motility and cell–cell interactions, a wound-healing assay was conducted. MDA cells were seeded into flat-bottomed 6-well plates in duplicate and cultured to form 80–90% confluent monolayer. Then, a wound was inflicted into the cell layer by using a sterile 200 µL pipette tip, and the cells were treated with 0, 7.5, or 12.5 µM DEA or 25 or 35 µM AM for up to 24 h. The wounds were imaged at 0, 6, and 24 h using an EVOS microscope (Thermo Scientific Hungary, Budapest, Hungary) at 4× magnification. The differences in distance were measured using ImageJ software v.1.53. The experiment was repeated 3 times.

### 4.6. Subcellular Fractionation

To study the signaling of specific proteins in different cell compartments, differential centrifugation was performed. Four partially confluent 10-cm plates of MDA cells were harvested for each DEA or AM concentration after 24 h of treatment (0, 7.5, or 12.5 µM DEA, or 25 or 35 µM AM). Plates were washed twice with 5 mL of PBS and resuspended in 1 mL of fractionation buffer (250 mM of sucrose, 20 mM of 2-[4-(2-hydroxyethyl)piperazin-1-yl] ethanesulfonic acid (HEPES), pH 7.4, 10 mM of KCl, 1.5 mM of MgCl_2_, 1 mM of ethylenediamine-tetraacetic acid (EDTA), 1 mM of ethyleneglycol-tetraacetic acid (EGTA), 1 mM of dithiothreitol (DTT), and protease and phosphatase inhibitor cocktails (Sigma, #P2714 Sigma-Aldrich, St. Louis, MO, USA). The cell lysates were homogenized using a potter homogenizer, cooled on ice, and centrifuged at 800× *g* for 15 min at 4 °C. The pellet containing the nucleus was resuspended in 1 mL of fractionation buffer, re-homogenized, and centrifuged at 500× *g* for 15 min at 4 °C. This step was repeated one more time with 1000× *g* for 15 min and then the pellet was resuspended in lysis buffer (10% glycerol, 25 mM of NaCl, 50 mM of NaF, 10 mM of Na-pyrophosphate, 2 nM of EGTA, 2 nM of DTT, 20 nM of p-nitrophenyl-phosphate, 25 mM of Tris-HCl, pH 7.4, 50 nM of beta-glycerophosphate, and 0.1% Triton X-100) to yield the nuclear fraction. The supernatants obtained by the centrifugation step at 800× *g* for 15 min were centrifuged at 11,000× *g* for 10 min at 4 °C. The supernatants and the pellet of this step yielded the cytoplasmic and mitochondrial fractions, respectively. Mitochondrial and nuclear fractions were sonicated 3 times for 10 s each.

### 4.7. Immunoblot Analysis

MDA-MB-231 cells were plated on 10 cm plates at a starting density of 10^6^ cell/plate, cultured overnight, and then treated with 0, 7.5, or 12.5 µM DEA, or 25 or 35 µM AM for 24 h. Following the treatment, the cells were harvested, washed twice with 1× PBS, and incubated in a chilled lysis buffer (0.5 mM of sodium–metavanadate, 1 mM of EDTA, and protease inhibitor cocktail (1:200)) on ice for 15 min with continuous shaking. Then, the samples were sonicated one time for 10 s and the lysates were boiled for 5 min, subjected to 10% or 12% sodium dodecyl sulfate polyacrylamide gel electrophoresis (SDS-PAGE), and transferred to nitrocellulose membranes. The membranes were then blocked in 5% bovine serum albumin (BSA) for 1.5 h at room temperature, followed by exposure to primary antibodies diluted in blocking solution at 4 °C overnight. Appropriate horseradish peroxidase-conjugated secondary antibodies were used at a dilution of 1:5000 (anti-mouse and anti-rabbit IgGs; Sigma-Aldrich, St. Louis, MO, USA). Chemiluminescence generated by applying the WesternBright ECL HRP substrate (Advansta, San Jose, CA, USA) was measured using an Azure 300 (Azure Biosystems, Dublin, CA, USA) high-resolution imaging system. Pixel volumes of the bands were determined using ImageJ software. For membrane stripping and re-probing, the membranes were washed in a stripping buffer containing 0.1 M glycine and 5 M MgCl_2_ (pH 2.8) for 30 min at room temperature. After washing and blocking, the membranes were re-probed as described above.

### 4.8. Bioenergetics Assay

To determine the rate difference between oxidative (mitochondrial ATP) and fermentative energy production, the oxygen consumption rate (OCR) and extracellular acidification rate (ECAR) were monitored using an Agilent Seahorse XFp Analyzer (Agilent, Santa Clara, CA, USA). MDA-MB-231 cells were seeded into 8-well XFp cell culture miniplates at a starting density of 2 × 10^4^ cells/well in duplicate. After overnight incubation, the cells were treated with 0, 7.5, or 12.5 µM DEA, or 25 or 35 µM AM for 6 hrs. After the treatment, the medium was replaced with Seahorse XF assay medium (Agilent, Santa Clara, CA, USA) at pH 7.4 supplemented with 10 mM glucose, 1 mM pyruvate, and 2 mM glutamine. We used the following inhibitors for the measurement: 1 µM oligomycin, 1 µM of FCCP, and 1 µM of rotenone/antimycin A. Non-cellular oxygen consumption was assessed in two wells running without cells and was subtracted from the corresponding OCR value. The OCR and ECAR data were normalized to the mg protein content determined using a DC Protein Assay kit (Bio-Rad, Marnes-la-Coquette, France). No other data correction was applied. The experiment was repeated twice in duplicate. 

### 4.9. ∆Ψ_m_ Assay 

To observe the effect on the mitochondrial membrane potential of DEA or AM-treated MDA cells, Rhodamine 123 (Rh123, Sigma-Aldrich, St. Louis, MO, USA) was used. MDA cells (5 × 10^5^ cells/well) were cultured onto 96-well plates for 24 h. Then, the cells were treated with 0, 7.5, or 12.5 µM DEA, or 25 or 35 µM AM for 6 h, rinsed with HBSS, and incubated with 1.5 µM Rh123 for 30 min in the dark. Subsequently, the cells were washed with HBSS. Images were taken using a Nikon microscope (Inverted Microscope Eclipse Ti-U Instruction, Auro-Science Ltd., Budapest, Hungary) equipped with a SPOT RT3 2 Mp Monochrome camera, including SPOT Advanced software (v.SPOT 5.2), using a 20× objective. For quantitative analysis, MDA cells were seeded onto 6-well plates and incubated to reach 70% confluency, followed by the treatment with the same drugs concentrations for 6 hrs. Then, the cells were washed with HBSS twice, trypsinized, and incubated with 1 µM Rh123 for 30 min in the dark. Prior to the measurement, the cells were washed twice with HBSS to remove the nonspecific staining. MFI of Rh123-labeled cells was measured using 10^4^ events per sample by SONY SH800 Cell Sorter (SONY Biotechnology, San Jose, CA, USA) with 488 nm excitation and 525/50 nm detection. Analysis was carried out with Cell Sorter Software v.2.1.6 (SONY Biotechnology, San Jose, CA, USA). All experiments were performed in triplicate.

### 4.10. Mitochondrial Permeability Transition Pore Opening (MPTP)

MPTP opening was determined using the MitoProbe Transition Pore Assay (Sigma-Aldrich, St. Louis, MO, USA) by the calcein/Co^2+^ quenching technique. Briefly, MDA-MB-231 cells were seeded onto 96-well plates (5 × 10^4^ cells/well) and incubated under standard conditions. Forty-eight hours later, the cells were treated with 0, 7.5, or 12.5 µM DEA, or 25 or 35 µM AM for 6 h. Then, the cells were washed once with HBSS, incubated with 2 µM calcein-AM, 0.5 mM cobalt chloride (CoCl_2_), and 30 µM Hoechst in the dark for 30 min. Prior to the measurement, the cells were washed twice with HBSS to remove the nonspecific staining. Images were taken using a Nikon microscope (Inverted Microscope Eclipse Ti-U Instruction, Hungary) equipped with a SPOT RT3 2 Mp monochrome camera, including SPOT Advanced software, using a 20× objective. The same microscopic field was first imaged using the red channel, followed by the green channels, and the resulting images were merged using Adobe Photoshop 7.0. In the control experiments, we did not observe considerable bleed-through between the red and green channels. The same calibration parameters were applied to the batch of images obtained from the same experiment. For quantification, ImageJ software was used after converting the images to greyscale. To compare fluorescence intensity among the differently treated groups, the corrected total cell fluorescence (CTCF) was calculated using the following equation: CTCF = Integrated Density − (area of selected cells × mean fluorescence of background readings). CFTF was normalized to the fluorescence intensity of Hoechst. All experiments were performed in triplicate.

### 4.11. Intracellular Calcium ([Ca^2+^]_i_)

Intracellular Ca^2+^ was measured with a Fluo-4-AM probe (Sigma-Aldrich, St. Louis, MO, USA). Briefly, MDA-MB-231 cells (5 × 10^4^ cells/well) were cultured onto 96-well plates and incubated under standard conditions. After achieving 60% cell confluency, the cells were treated with 0, 7.5, or 12.5 µM DEA, or 25 or 35 µM AM in media supplemented with 3 mM of calcium for 3 h. Then, the cells were washed twice with HBSS, and incubated with a mixture of 2.5 µM Fluo-4-AM and 30 µM Hoechst for 30 min in the dark. Prior to the measurement, the cells were washed twice with HBSS to remove the nonspecific staining. Images were taken using a Nikon microscope (Inverted Microscope Eclipse Ti-U Instruction, Hungary) equipped with a SPOT RT3 2 Mp monochrome camera, including SPOT Advanced software, using a 20× objective. Mean fluorescence intensity (MFI) was detected using 10^4^ events per sample by SONY SH800 Cell Sorter (SONY Biotechnology, San Jose, CA, USA) with 488 nm excitation and 525/50 nm detection. All experiments were performed in triplicate.

### 4.12. D Spheroids 

MDA-MB-231 cells (8 × 10^4^ cells/well) were seeded in U-shaped, polyhydroxyethylmethacrylate polymer (poly-HEMA)-coated 96-well plates and were maintained under appropriate conditions for four days. Then, spheroids were treated with 0, 7.5, or 12.5 µM DEA or 25 or 35 µM AM for 72 h. Following the treatment, spheroids were stained with a mixture of 2 µM calcein-AM and 3 µM of ethidium homodimer (EthD-1, Sigma-Aldrich, St. Louis, MO, USA) for 1 h. Prior to taking the images, the wells were washed twice to remove the nonspecific staining. Images were taken using a Nikon microscope (Inverted Microscope Eclipse Ti-U Instruction, Hungary) equipped with a SPOT RT3 2 Mp Monochrome camera, including SPOT Advanced software, using a 10× objective. To compare fluorescence intensity among different spheroids, CTCF was calculated using ImageJ v.1.53. The experiment was repeated three times.

### 4.13. Analysis of Mitochondrial Network Dynamics

MDA-MB-231 cells were seeded on glass coverslips and were cultured overnight. The cells were treated with 0, 7.5, or 12.5 µM DEA, or 25 or 35 µM AM, rinsed twice in PBS, and incubated in PBS containing 50 nM of MitoTracker Red for 30 min in a CO_2_ incubator at 37 °C. Fluorescence images were taken via the 60× objective of a Nikon microscope (Inverted Microscope Eclipse Ti-U Instruction, Hungary) equipped with a SPOT RT3 2 Mp monochrome camera. Quantitative determination of mitochondrial fragmentation was performed as described. Mitochondria shorter than 2 μm were considered fragmented, while those longer than 5 μm were considered filamentous. All experiments were performed in triplicate.

### 4.14. Statistical Analysis

The results are shown as means ± SD (Figure 6) or means ± SEM. ANOVA using the post hoc Dunnett test was employed to calculate the concentration-dependent effects of AM or DEA in each experiment. Statistical analyses were performed using IBM SPSS Statistics v20.0. Differences at *p* < 0.05 were regarded as significant.

## 5. Conclusions

Regardless of their precise molecular mechanisms, AM and DEA have been shown to possess the characteristics of mitochondrial drugs and can be considered potential candidates for cancer therapy. Although triple-negative breast cancers may differ in their growth and drug-sensitivity characteristics from other cancer types [63], we previously demonstrated that the mitochondrial effects of DEA significantly contributed to its antineoplastic effects in various human cancer lines [19,20,21,22,23]. Accordingly, our findings on the MDA-MB-231 cancer cell line may be relevant for several other cancer types as well, especially with regard to mitochondrial effects and energetic considerations.

## 6. Patents

Patent numbers: US9884040B2; EP3173081.

## Data Availability

Data are contained within the article.

## Abbreviations

7-AAD	7-aminoactinomycin D
AM	Amiodarone
Bax	Bcl-2-like protein 4
BC	Breast cancer
Bcl2	B-cell lymphoma2
BH3	Bcl homology domain-3 protein
DEA	Desethylamiodarone
ΔΨ_m_	Mitochondrial membrane potential
DRP1	Dynamin-related protein 1
ECAR	Extracellular acidification rate
EMT	Epithelial-to-mesenchymal transition
FCCP	Carbonyl cyanide 4-(trifluoromethoxy) phenylhydrazone
Fis1	Fission 1 protein
FITC	Fluorescein isothiocyanate
Her2	Human epidermal growth factor receptor 2
HR+	Hormone receptor positive
MFN	Mitofusin
OCR	Oxygen consumption rate
OD	Optical density
Opa1	Optic atrophy1
PARP	Poly(ADP-ribose) polymerase
PBS	Phosphate-buffered saline
ROS	Reactive oxygen species
SD	Standard deviation
SEM	Standard error of the mean
SRB	Sulforhodamine B
TN	Triple negative
